# Comparison between blood hemoglobin concentration determined by point-of-care device and complete blood count in adult patients with dengue

**DOI:** 10.1371/journal.pntd.0009692

**Published:** 2021-08-16

**Authors:** Kantasit Wisanuvej, Kochawan Boonyawat, Chutchaiwat Savetamornkul, Sirapong Virapongsiri, Jatupon Krongvorakul, Somnuek Sungkanuparph, Angsana Phuphuakrat

**Affiliations:** 1 Department of Medicine, Faculty of Medicine Ramathibodi Hospital, Mahidol University, Bangkok, Thailand; 2 Chakri Naruebodindra Medical Institute, Faculty of Medicine Ramathibodi Hospital, Mahidol University, Samut Prakan, Thailand; 3 Department of Pathology, Faculty of Medicine Ramathibodi Hospital, Mahidol University, Bangkok, Thailand; George Washington University School of Medicine and Health Sciences, UNITED STATES

## Abstract

**Background:**

Hematocrit measurement has been an indispensable tool for monitoring plasma leakage and bleeding in dengue patients. However, hematocrit measurement by automated methods is hampered by frequent venipunctures. Utility of point-of-care hemoglobin (POC-Hb) test for monitoring dengue patients has not been established. We evaluated the relationship between hemoglobin measured by POC-Hb testing and hematocrit measured by the automated method in adult dengue patients.

**Methodology and principal findings:**

Adult dengue patients were recruited at two university hospitals in Thailand from October 2019 to December 2020. POC-Hb test was performed using capillary blood simultaneously with venipuncture to obtain whole blood for an automated complete blood count (CBC) analysis. The correlation of hemoglobin and hematocrit measurement was evaluated. A total of 44 dengue patients were enrolled. Twenty-nine patients (65.9%) were female, with a median age of 31 years (interquartile range 22–41). Of the enrolled patients, 30 (68.2%), 11 (25.0%), and 3 (6.8%) were classified as dengue without warning signs, with warning signs, and severe dengue, respectively. Seven patients (15.9%) had hemoconcentration, and five patients (11.3%) had bleeding. A total of 216 pairs of POC-Hb and CBC were evaluated. A significant positive correlation was observed between hemoglobin measured by POC-Hb testing and hematocrit measured by an automated CBC (*r* = 0.869, p <0.001). Bland-Altman analysis between hemoglobin measured by POC-Hb testing and an automated CBC showed a bias of -0.43 (95% limit of agreement of -1.81 and 0.95). Using the cutoff of POC-Hb ≥20% as a criteria for hemoconcentration, the sensitivity and specificity of hemoconcentration detected by POC-Hb device were 71.4% and 100.0%, respectively.

**Conclusions:**

Hemoglobin measurement by POC-Hb testing has a strong correlation with hematocrit in adult patients with dengue fever. However, the sensitivity in detecting hemoconcentration is fair. The adjunct use of capillary POC-Hb testing can decrease the frequency of venipuncture. Further study in children is encouraged.

## Author summary

The natural history of dengue has the unique characteristic of plasma leakage that results in hemoconcentration as well as vasculopathy and thrombocytopenia that result in bleeding. Therefore, hematocrit monitoring is a crucial tool to guide management in dengue patients. Microhematocrit adjunct with a complete blood count (CBC) to monitor dengue patients were traditionally performed to decrease the frequency of venipuncture. However, microhematocrit is currently less preferred to the point-of-care hemoglobin monitoring tools to measure red cells in other diseases. There has been no study investigating the feasibility of using point-of-care hemoglobin (POC-Hb) to monitor dengue patients. We studied the correlation of hemoglobin measured by POC-Hb testing and hematocrit measured by an automated CBC in adult dengue patients. We were able to show a strong positive correlation between the two measurements (*r* = 0.869, p <0.001). Using the rise of POC-Hb value of ≥20% above the convalescent value as the criteria for detecting hemoconcentration by POC-Hb testing, measurement of hemoglobin using POC-Hb has a sensitivity of 71.4% for detecting hemoconcentration compared to the traditional hematocrit criteria. Our findings support the use of POC-Hb testing adjunct with a complete blood count to guide fluid management in dengue patients.

## Introduction

Dengue is a mosquito-borne viral infection that is mainly found in tropical and sub-tropical climates [1]. Dengue can cause a significant impact not only in terms of health burden but also an economic and social burden on the affected populations of endemic regions. A broad clinical spectrum of dengue has been recognized, and its clinical evolution and outcome are difficult to predict. While most dengue produces a self-limiting mild clinical course, it can potentially progress to severe disease with possible fatal complications. Plasma leakage either with or without hemorrhage is a critical feature that indicates severe dengue [2]. For practical reasons, it has been suggested to classify given individuals with non-severe dengue into dengue with or without warning signs. The warning signs include a mucosal bleed, clinical fluid accumulation, and increased hematocrit with a rapid decrease in platelet count [2].

In dengue, an increased hematocrit is a clue for plasma leakage, whereas a decreased hematocrit is a clue for bleeding. Therefore, hematocrit monitoring is crucial to guide clinicians to manage dengue patients properly [2,3]. Hematocrit is the proportion of red blood cells by volume in the blood [4]. Hematocrit monitoring in dengue patients has traditionally been performed by microhematocrit on capillary blood, adjunct with venous blood measured by an automated complete blood count (CBC). Microhematocrit can be determined by centrifuging the capillary tubes filled with blood to pack the red cells while minimizing trapped extracellular fluid. The advantage of using microhematocrit adjunct with a CBC is to reduce the invasiveness of frequent venipunctures. This method is reliable and simple [5]. However, as a manual method, it would carry a risk of injury to an operator and a chance of less accurate results compared with an automated CBC.

Currently, there has been the use of point-of-care hemoglobin (POC-Hb) for hemoglobin measurement. Many studies have reported the clinical use of POC-Hb in various settings, including blood donation [6–8] and anemia screening [7], hemoglobin monitoring during cardiac operation [9], and hemoglobin measurement in intensive care unit patients [10,11]. Hemoglobin is a protein in red blood cells and functions in oxygen delivery to the tissues. For an automated CBC, hemoglobin concentration is determined by the photometric method [12]. Measurement of POC-Hb is accessed in a similar fashion but uses capillary blood inserted into the microcuvette or cartridge of the machine. In the HemoCue system, hemoglobin is converted to azidemethemoglobin in the microcuvette, and the absorbance can be estimated at two wavelengths [13]. POC-Hb is thus considered a convenient, rapid, and minimally invasive method to evaluate hemoglobin.

Many previous studies demonstrated that hemoglobin is associated with the severity of dengue. Hemoglobin in dengue patients with plasma leakage was higher than in those without leakage [14]. Also, a study in children with dengue found that an increase in hemoglobin and hematocrit was associated with dengue disease severity [15]. However, the investigation regarding the use of POC-Hb for monitoring plasma leakage and bleeding in dengue patients is lacking. We aimed to evaluate the relationship between hemoglobin measured by POC-Hb testing and hematocrit measured by an automated CBC in adult dengue patients and to study if POC-Hb testing could detect hemoconcentration.

## Methods

### Ethics statement

The study protocol was reviewed and approved by the Ethical Clearance Committee on Human Rights Related to Research Involving Human Subjects, Faculty of Medicine Ramathibodi Hospital, Mahidol University (COA. MURA2019/777). Written consent was obtained from the participants.

### Patients and study design

A prospective cohort of dengue patients was conducted at Ramathibodi Hospital in Bangkok, and Chakri Naruebodindra Medical Institute, in Samut Prakan, Thailand, from October 2019 to December 2020. We included adult patients aged 18 years or older who were diagnosed with dengue infection. Dengue infection was defined by acute fever together with a positive result(s) of dengue tests, including (1) dengue NS1 antigen (SD Biosensor, Gyeonggi-do, Korea), (2) dengue polymerase chain reaction (Vitassay, Huesca, Spain), or (3) dengue IgM (SD Biosensor, Gyeonggi-do, Korea) with thrombocytopenia. Patients who had conditions that may affect red blood cell counts, such as rouleaux formation, agglutination, and hemolytic anemia, were excluded.

### Data collection and procedures

Patients’ demographic data, clinical manifestations, such as duration of fever, dengue severity, bleeding, and laboratory data, such as CBC and liver function tests, were collected. The attending physicians ordered a serial CBC according to their decision for the routine management of dengue patients. Venipuncture was performed by hospital staff (phlebotomists or nurses). A CBC was measured by Mindray BC-6800Plus (Mindray, Shenzhen, China) at Ramathibodi Hospital, and Sysmex XN-3000 (Sysmex, Hyogo, Japan) at Chakri Naruebodindra Medical Institute. POC-Hb testing was performed on capillary blood and read by the HemoCue Hb 201^+^ (HemoCue AB, Ängelholm, Sweden). POC-Hb testing was conducted by three research physicians who were trained on the use of the device. These two procedures were performed simultaneously until the patient in an inpatient setting was discharged. In an outpatient setting, POC-Hb testing was performed simultaneously with a CBC at each visit until recovery.

### Hemoconcentration identification

Hemoconcentration was defined by an increase of ≥20% in hematocrit compared with a convalescent value [2]. Since there is no definition of hemoconcentration using hemoglobin level, we have extrapolated from the traditional alteration in hematocrit level. Therefore, for the use of POC-Hb testing, we defined a rise of ≥20% of POC-Hb compared with a convalescent value of hemoglobin measured by an automated CBC (laboratory hemoglobin; laboratory Hb) as hemoconcentration. Sensitivity, specificity, positive predictive value (PPV), negative predictive value (NPV) [16], and a 95% confidence interval (CI) were calculated to assess the performance of POC-Hb device for detecting hemoconcentration compared to the traditional hematocrit criteria.

### Statistical analysis

Continuous variables were presented as medians and interquartile ranges (IQR), whereas categorical variables were presented as frequencies and percentages. Correlations between hematocrit and hemoglobin from various measurements were determined by Pearson’s correlation. The Bland-Altman analysis was performed to evaluate the agreement of laboratory Hb and POC-Hb. Differences between each pair of measurements (POC-Hb—laboratory Hb) were plotted on the vertical axis against the averages of the pair (POC-Hb + laboratory Hb)/2 on the horizontal axis [17]. Bias was the difference of results between POC-Hb and laboratory Hb measurements. Logistic regression was used to determine the factors associated with the discordance between hemoglobin values measured by the POC-Hb device and automated CBC. Variables that presented a p-value <0.2 from univariate logistic regression were considered in a multivariate logistic regression model. A p-value of <0.05 was considered statistically significant. The receiver operating characteristic (ROC) curve was analyzed to study the POC-Hb test performance and its cutoff for maximum sensitivity and specificity in detecting hemoconcentration compared to the traditional hematocrit criteria [18]. Statistical analyses were performed by Stata statistical software version 16.0 (StataCorp, College Station, TX, USA) and Prism GraphPad version 9.1.2 (GraphPad Software, San Diego, CA, USA).

## Results

### Patients

A total of 44 dengue patients were recruited. The characteristics of the patients are shown in Table 1. Twenty-nine patients (65.9%) were female. The median (interquartile range; IQR) age of patients was 31 (22–41) years, and the median (IQR) body mass index was 23.7 (20.9–26.3) kg/m^2^. The majority (68.2%) of the patients were dengue without warning signs, 25.0% were dengue with warning signs, and 6.8% severe dengue. Of the severe dengue patients, two patients had elevated aspartate aminotransferase more than 1,000 U/L without signs of severe plasma leakage, and another patient had hypotension before entering the study. Almost all patients (93.2%) were hospitalized, and the median duration of hospital stay was 4.5 days. Twenty-one patients (47.7%) and 23 patients (52.3%) were receiving care at Ramathibodi Hospital and Chakri Naruebodindra Medical Institute, respectively. The medians of hemoglobin and hematocrit at dengue presentation were 13.8 g/dL and 41.6%, respectively. The medians of mean corpuscular volume (MCV) and platelets were 87.0 fL and 98,500/mm^3^, respectively. Seven patients (15.9%) had hemoconcentration, and five patients (11.3%) had hemorrhage (i.e., menstruation, epistaxis, and upper gastrointestinal bleeding). No patients developed severe plasma leakage [2] during the study. Characteristics of the patients stratified by disease severity are shown in S1 Table.

**Table 1:** Characteristics of 44 dengue patients.

Characteristic	n = 44
Female gender [n (%)]	29 (65.9)
Age (years) [median (IQR)]	31 (22–41)
BMI (kg/m^2^) [median (IQR)]	23.7 (20.9–26.3)
Underlying diseases [n (%)]	
- Diabetes mellitus	2 (4.6)
- Hypertension	5 (11.4)
Severity [n (%)]	
- Without warning signs	30 (68.2)
- With warning signs	11 (25.0)
- Severe	3 (6.8)
Day of fever onset (days) [median (IQR)]	3.0 (2.0–3.0)
Duration of hospital stay (days) [median (IQR)]	4.5 (3.0–5.0)
Laboratory values at presentation	
- Hemoglobin (g/dL) [median (IQR)]	13.8 (12.9–14.6)
- Hematocrit (%) [median (IQR)]	41.6 (28.9–44.6)
- MCV (fL) [median (IQR)]	87.0 (83.7–89.9)
White blood cell count (cells/mm^3^) [median (IQR)]	3,370 (2,650–5,215)
Platelet count (/mm^3^) [median (IQR)]	98,500 (63,500–150,500)
- AST (U/L) [median (IQR)]	72 (51–114)
- ALT (U/L) [median (IQR)]	37 (26–73)
Hemoconcentration [n (%)]	7 (15.9)
Hemorrhage [n (%)]	5 (11.3)
Inpatient [n (%)]	41 (93.2)

Abbreviations: ALT, alanine aminotransferase; AST, aspartate aminotransferase; BMI, body mass index; IQR, interquartile range; MCV, mean corpuscular volume

### Correlation and agreement of the testing

**Fig 1:**
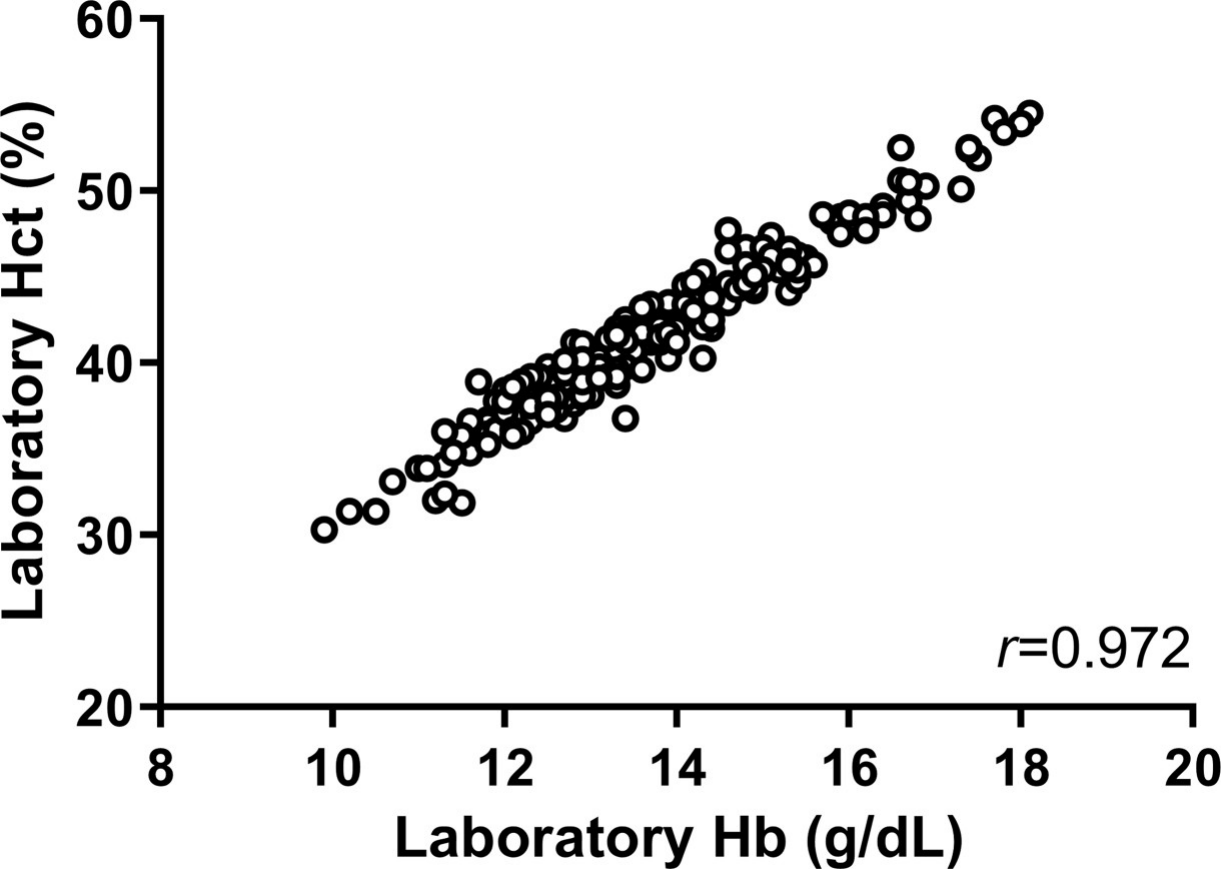
Scatter plot of laboratory hemoglobin and hematocrit.

The total CBC and POC-Hb evaluations of dengue patients in this cohort were 259 and 216 times, respectively. The median (IQR) of CBC evaluation was 5 (4–8) times/patient, and that of POC-Hb was 4 (3–7) times per patient. Some POC-Hb tests were not performed due to time constraint of the research physicians. Fig 1 shows the scatter plot of hemoglobin and hematocrit measured by an automated CBC. The laboratory Hb strongly correlated with hematocrit (*r* = 0.972; p <0.001).

**Fig 2:**
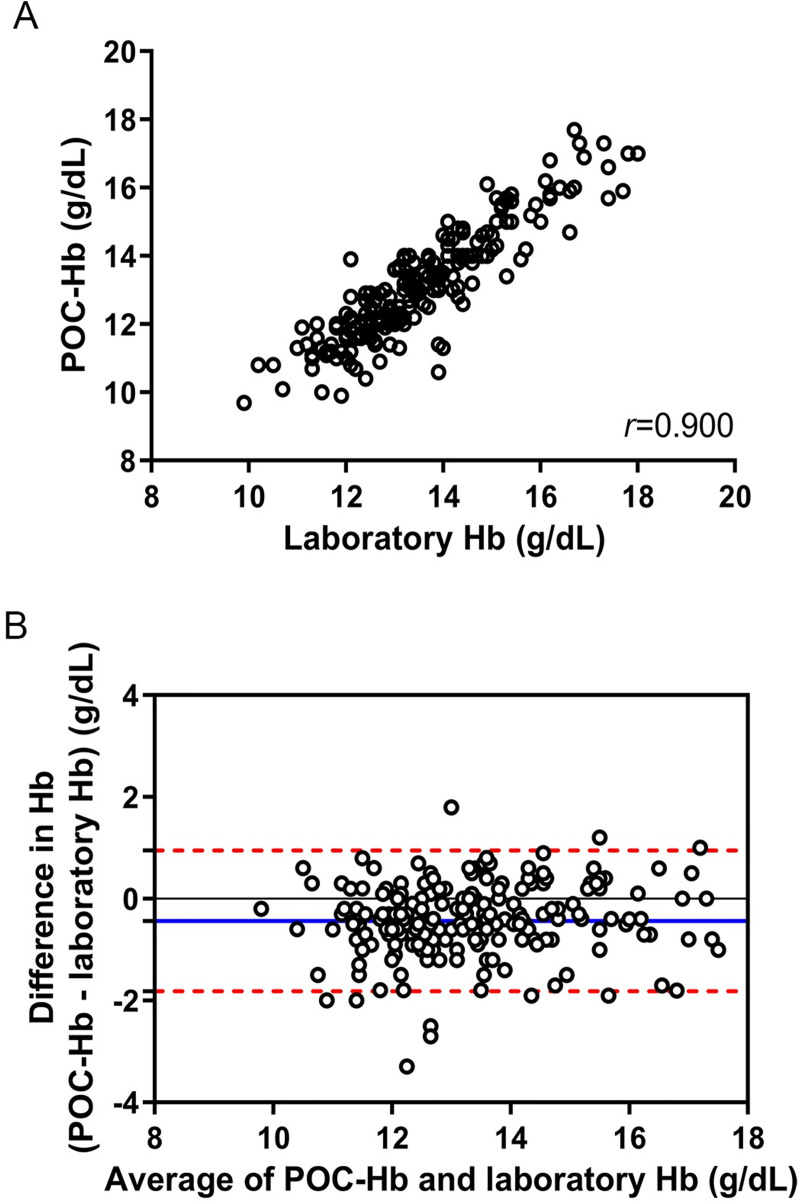
Scatter plot (A) and agreement (B) of point-of-care hemoglobin and laboratory hemoglobin. Blue line shows mean difference; upper and lower red dashed lines denote 95% upper and lower limits, respectively.

**Fig 3:**
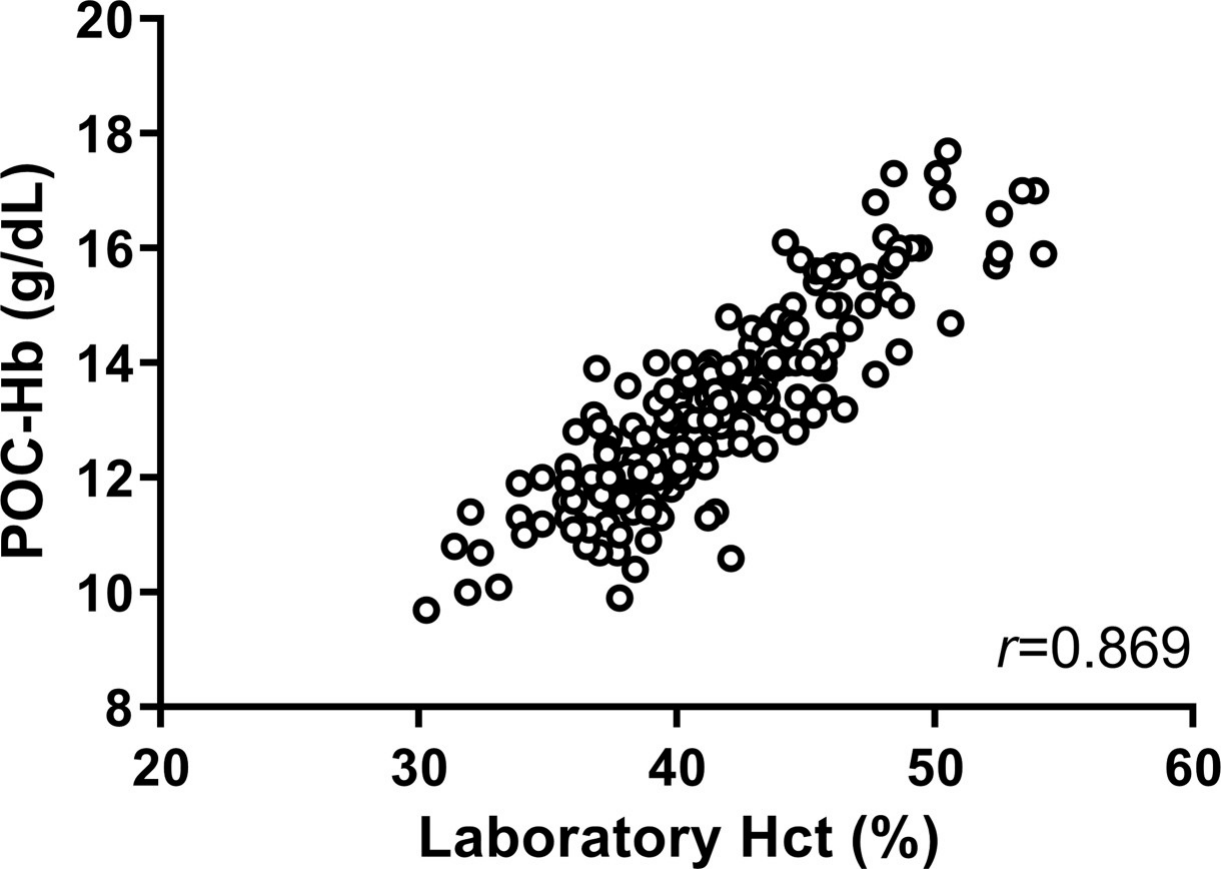
Scatter plot of point-of-care hemoglobin and laboratory hematocrit.

The values of POC-Hb and laboratory Hb were significantly correlated, as shown in Fig 2A (*r* = 0.900; p <0.001). Agreement between POC-Hb and laboratory Hb was evaluated (Fig 2B). The bias ± standard deviation between POC-Hb and laboratory Hb was -0.43±0.70 g/dL, and the 95% limits of agreement were -1.82 to 0.95. There were 31 pairs of the test (14.4%) that showed more than ±1 g/dL difference between POC-Hb and laboratory Hb values. Factors including age, gender, disease severity, hospital, research physician, hemoconcentration, hemorrhage, hematocrit, and MCV at the presentation were included in logistic regression analysis for association with the discordance. However, multivariate logistic regression failed to identify factors associated with the discordance between hemoglobin values measured by the two methods (S2 Table). The correlation between POC-Hb and hematocrit was further evaluated (Fig 3), which demonstrated a significant correlation (*r* = 0.869; p <0.001). A subgroup analysis of the correlation between POC-Hb and hematocrit in the two institutes is shown in S1 Fig. A subgroup analysis of the correlations between hemoglobin and hematocrit, and the agreement of hemoglobin testing between the two methods stratified by disease severity proved a significant correlation in all subgroups (S2 Fig, p <0.001, all).

### Hemoconcentration identification by POC-Hb device

**Fig 4:**
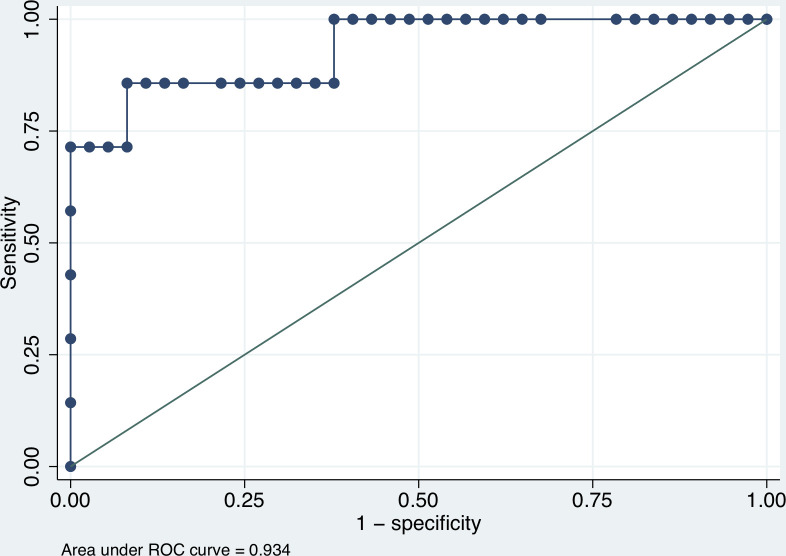
Receiver operating characteristic (ROC) curves for detecting hemoconcentration using a POC-Hb device compared with the traditional hematocrit criteria among dengue patients recruited at Ramathibodi Hospital and Chakri Naruebodindra Medical Institute.

In this study, seven patients (15.9%) had hemoconcentration by using the traditional hematocrit criteria. Using the traditional hematocrit criteria as a gold standard, POC-Hb device had a sensitivity, specificity, PPV, and NPV of 71.4, 100.0, 100.0, and 94.9%, respectively, for hemoconcentration identification. ROC curve analysis demonstrated a good performance of using POC-Hb value in detecting hemoconcentration (AUC = 0.93, 95% CI 0.83–1.00) (Fig 4). At the optimal cutoff value of 13.1%, POC-Hb device had a sensitivity of 85.7%, and a specificity of 91.9% for detecting hemoconcentration.

## Discussion

Hemoglobin measurement by POC-Hb has been studied for various purposes, mainly for the screening of anemia [6–11]. Nevertheless, there has been no published study using POC-Hb testing to monitor plasma leakage or bleeding in dengue patients. We demonstrated the strong positive correlation [19] between hemoglobin values measured by POC-Hb testing and hematocrit measured by an automated CBC.

Dengue has a unique pathogenesis of the increase in capillary permeability due to endothelial dysfunction, which results in plasma leakage [20]. A rise in hematocrit is an indication that plasma leakage has occurred, and proper fluid management is required. In dengue, vasculopathy and thrombocytopenia might also contribute to bleeding. A drop in hematocrit is a clue that guides a clinician to look for the bleeding. In the convalescent phase, plasma leakage reabsorption occurs. Therefore, hematocrit monitoring is a necessary procedure in dengue patients [2,3].

The traditional monitoring of hematocrit in dengue patients was the use of microhematocrit adjunct with an automated CBC. Although microhematocrit has the advantages of simplicity, is inexpensive, and the requirement of a small volume of capillary blood, it has some disadvantages including the fragile glass tube, which might lead to an occupational injury, and the possibility of blood loss during centrifugation [21]. Currently, there have been many tools for hemoglobin monitoring. However, none has been evaluated for dengue monitoring. In this study, we demonstrated the strong positive correlation of hemoglobin and hematocrit measured by automated CBC, and the strong positive correlation of hemoglobin measured by POC-Hb testing and hematocrit measured by automated CBC in adult dengue patients.

The agreement of hemoglobin between POC-Hb testing and laboratory Hb showed a bias of -0.43 g/dL suggesting the lower sensitivity for the use of POC-Hb for detecting hemoconcentration, and this was confirmed by the sensitivity of the test. Our study showed consistent findings regarding the test agreement between POC-Hb and laboratory Hb [22–25]. The overall mean difference of hemoglobin measured by HemoCue and an automated CBC in the systematic review and meta-analysis was 0.08 g/dL (95% limits of agreement -1.3 to 1.4 g/dL) [26]. However, most of the studies evaluated the two devices’ agreement of hemoglobin testing using the same blood sample (arterial or venous blood). Our study simulated the real-life situation for dengue patients that capillary blood was used adjunct with venous blood. Interestingly, Seguin and coworkers reported edema as the causal factor for discordance between capillary POC-Hb device and venous laboratory Hb measurements [27]. However, no patients developed edema in our study. We could not identify factors associated with the discordance.

From the ROC curve, the optimal cutoff value to detect hemoconcentration was an increased POC-Hb value of 13.1%. Therefore, the increase or decrease in POC-Hb values of approximately 1.0 g/dL might be appropriate to prompt the CBC evaluation of hemoconcentration or hemorrhage in dengue patients. This value corresponded to the traditional use of an increase or decrease in hematocrit value of 10% in practice that triggers the appropriate management in dengue patients [3].

To our knowledge, our study is the first study evaluating the efficacy of the use of POC-Hb testing in adult dengue patients. The strengths of this study include (1) CBC and POC-Hb tests were simultaneously conducted at most time points, and (2) the research physicians who performed POC-Hb testing were blinded to the CBC results. We accepted the limitations that (1) two different CBC analyzers were used in the two institutes, and (2) the limited number of patients, especially the severe dengue patients whose capillary POC-Hb value might have been affected by poor perfusion of capillary blood. However, the tests were performed on many time points during the course of the disease in each patient, which made sufficient paring for the comparison.

In conclusion, hemoglobin measurement using POC-Hb testing adjunct with CBC monitoring can be used to monitor plasma leakage as well as hemorrhage in dengue patients. The use of POC-Hb testing is less invasive and requires less blood compared to venipuncture for CBC evaluation. The study in the children population, which is another significant burden of dengue, is encouraging.

## Supporting information

S1 Table Characteristics of the dengue patients stratified by disease severity. (DOCX)

S2 Table Univariate and multivariate analysis of factors associated with the discordance of POC-Hb and laboratory Hb. (DOCX)

S1 Fig Scatter plot of point-of-care hemoglobin and laboratory hematocrit performed at Ramathibodi Hospital (A) and Chakri Naruebodindra Medical Institute (B).
(TIFF)

S2 Fig Subgroup analysis of correlation between laboratory hemoglobin and hematocrit measured by an automated CBC (left panels), agreement of point-of-care hemoglobin and laboratory hemoglobin (middle panels), and correlation between point-of-care hemoglobin and laboratory hematocrit (right panels) in dengue patients without warning signs (A, D, G), dengue patients with warning signs and severe dengue (B, E, H), and dengue patients who had hemoconcentration (C, F, I).
(TIFF)

S1 Data Data used for the analysis. (XLSX)
