# Supplementary material for: Comparison between blood hemoglobin concentration determined by point-of-care device and complete blood count in adult patients with dengue
Source: PLoS Negl Trop Dis. 2021 Aug 16;15(8):e0009692. doi: 10.1371/journal.pntd.0009692 (PMC8389841; doi:10.1371/journal.pntd.0009692)
Supplement: S1 Table — (DOCX) [file pntd.0009692.s001.docx]

**S1 Table.** Characteristics of the dengue patients stratified by disease severity.

|  | Dengue without warning signs  (n=30) | Dengue with warning signs and severe dengue  (n=14) | p-value |
| --- | --- | --- | --- |
| Female gender [n (%)] | 22 (73.3) | 7 (50.0) | 0.177 |
| Age (years) [median (IQR)] | 32 (24-38) | 29 (20-45) | 0.791 |
| BMI (kg/m^2^) [median (IQR)] | 23.6 (21.0-26.7) | 23.9 (20.8-25.0) | 0.960 |
| Underlying diseases [n (%)] |  |  |  |
| - Diabetes mellitus | 0 (0.0) | 2 (14.3) | 0.096 |
| - Hypertension | 2 (6.7) | 3 (21.4) | 0.307 |
| Day of fever onset (days) [median (IQR)] | 3 (2-3) | 3 (2-3) | 0.472 |
| Duration of hospital stay (days) [median (IQR)] | 4 (3-5) | 5 (4-6) | 0.027 |
| Laboratory values at presentation |  |  |  |
| - Hemoglobin (g/dL) [median (IQR)] | 13.7 (12.5-14.4) | 14.2 (13.3-14.8) | 0.104 |
| - Hematocrit (%) [median (IQR)] | 40.8 (38.5-43.8) | 44.0 (41.1-47.7) | 0.044 |
| - MCV (fL) [median (IQR)] | 87.0 (82.7-88.8) | 88.2 (86.1-93.2) | 0.134 |
| - White blood cell count (cells/mm^3^) [median (IQR)] | 3,320 (2,420-5,200) | 3,650 (2,700-5,400) | 0.392 |
| - Platelet count (/mm^3^) [median (IQR)] | 103,500  (75,000-175,000) | 67,000  (38,000-127,000) | 0.041 |
| - AST (U/L) [median (IQR)] | 72 (51-107) | 78 (35-244) | 0.660 |
| - ALT (U/L) [median (IQR)] | 37 (21-65) | 39 (27-145) | 0.518 |
| Hemoconcentration [n (%)] | 0 (0.0) | 7 (50.0) | <0.001 |
| Hemorrhage [n (%)] | 3 (10.0) | 2 (14.3) | 0.647 |

Abbreviations: ALT, alanine aminotransferase; AST, aspartate aminotransferase; BMI, body mass index; IQR, interquartile range; MCV, mean corpuscular volume
