# Supplementary material for: Comparison between blood hemoglobin concentration determined by point-of-care device and complete blood count in adult patients with dengue
Source: PLoS Negl Trop Dis. 2021 Aug 16;15(8):e0009692. doi: 10.1371/journal.pntd.0009692 (PMC8389841; doi:10.1371/journal.pntd.0009692)
Supplement: S2 Table — (DOCX) [file pntd.0009692.s002.docx]

**S2 Table.** Univariate and multivariate analysis of factors associated with the discordance of POC-Hb and laboratory Hb.

| **Factors** | **Univariate analysis** | | | **Multivariate analysis** | | |
| --- | --- | --- | --- | --- | --- | --- |
|  | **OR** | **95% CI** | **p-value** | **OR** | **95% CI** | **p-value** |
| Age | 1.01 | (0.97-1.06) | 0.562 |  |  |  |
| Male gender | 0.82 | (0.23-2.85) | 0.751 |  |  |  |
| Study hospital | 0.83 | (0.25-2.73) | 0.763 |  |  |  |
| Research physician | 0.87 | (0.42-1.80) | 0.713 |  |  |  |
| Dengue with warning signs and severe dengue | 3.75 | (0.95-14.76) | 0.059 | 3.00 | (0.71-12.69) | 0.135 |
| Hemoconcentration | 2.94 | (0.50-17.14) | 0.230 |  |  |  |
| Hemorrhage | 4.67 | (0.48-45.62) | 0.185 | 4.88 | (0.48-49.94) | 0.476 |
| Hematocrit* | 1.04 | (0.90-1.19) | 0.607 |  |  |  |
| MCV* | 1.10 | (0.96-1.26) | 0.153 | 1.09 | (0.71-12.69) | 0.267 |

*Values at the presentation

Abbreviations: CI, confidence interval; MCV, mean corpuscular volume; OR, odds ratio
