# Supplementary figures and images for: Comparison between blood hemoglobin concentration determined by point-of-care device and complete blood count in adult patients with dengue
Source: PLoS Negl Trop Dis. 2021 Aug 16;15(8):e0009692. doi: 10.1371/journal.pntd.0009692 (PMC8389841; doi:10.1371/journal.pntd.0009692)

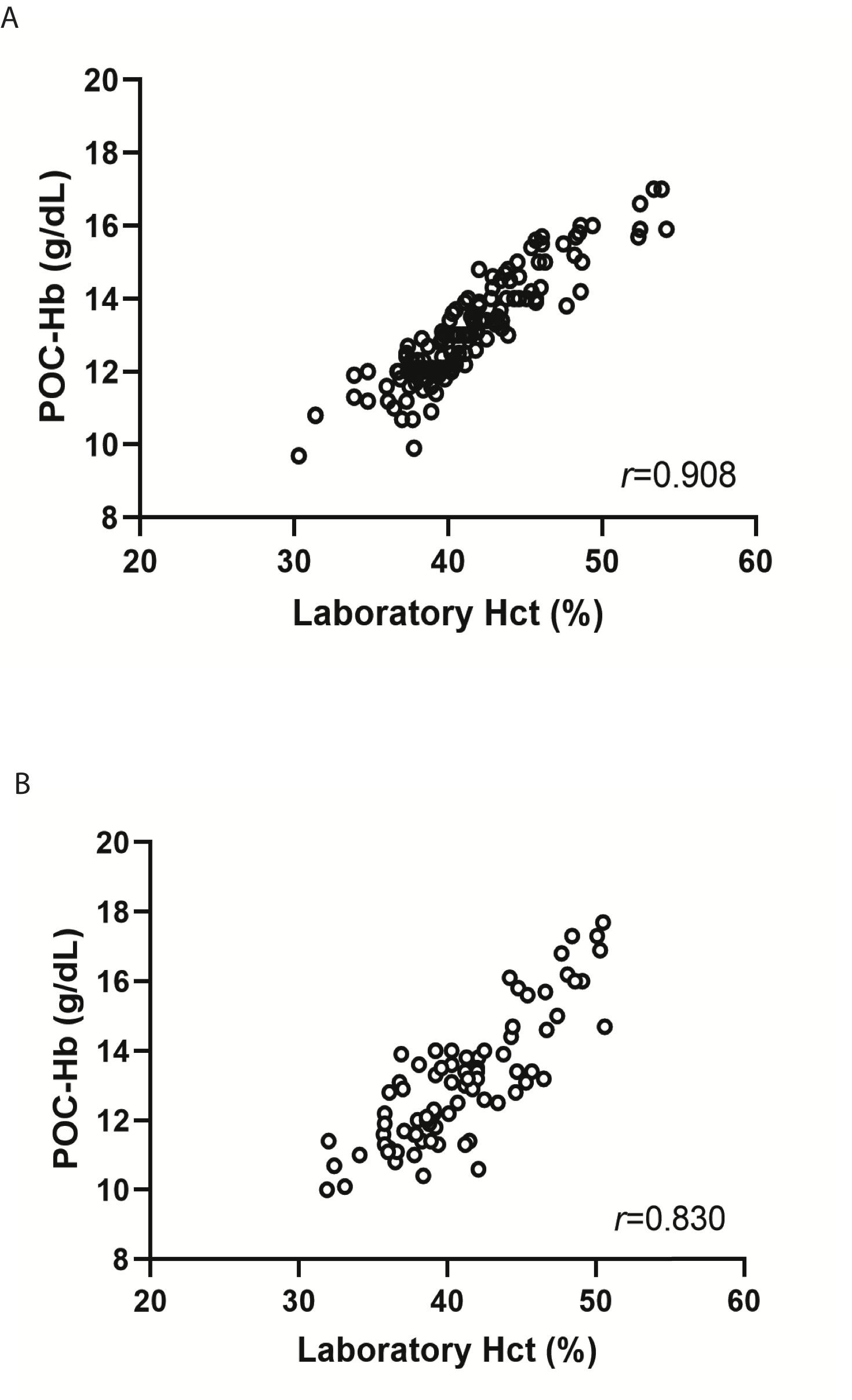

Supplement: S1 Fig — Scatter plot of point-of-care hemoglobin and laboratory hematocrit performed at Ramathibodi Hospital (A) and Chakri Naruebodindra Medical Institute (B). (TIFF) [file pntd.0009692.s003.tiff]
